# The *Helicobacter pylori* CagY Protein Drives Gastric Th1 and Th17 Inflammation and B Cell Proliferation in Gastric MALT Lymphoma

**DOI:** 10.3390/ijms22179459

**Published:** 2021-08-31

**Authors:** Chiara Della Bella, Maria Felicia Soluri, Simone Puccio, Marisa Benagiano, Alessia Grassi, Jacopo Bitetti, Fabio Cianchi, Daniele Sblattero, Clelia Peano, Mario Milco D’Elios

**Affiliations:** 1 Department of Experimental and Clinical Medicine, University of Florence, 50134 Florence, Italy; chiara.dellabella@unifi.it (C.D.B.); marisa.benagiano@unifi.it (M.B.); alessia.grassi@unifi.it (A.G.); jacopo.bitetti@gmail.com (J.B.); fabio.cianchi@unifi.it (F.C.); 2 Department of Health Sciences & IRCAD, Università del Piemonte Orientale, 28100 Novara, Italy; mariafelicia.soluri@med.uniupo.it; 3 Center for Translational Research on Autoimmune and Allergic Disease, Università del Piemonte Orientale, 28100 Novara, Italy; 4 Genomic Unit, IRCCS, Humanitas Clinical and Research Center, 20090 Milan, Italy; simone.puccio@humanitasresearch.it; 5 Department of Life Sciences, University of Trieste, 34127 Trieste, Italy; dsblattero@units.it; 6 Institute of Genetic and Biomedical Research, UoS Milan, National Research Council, 20090 Milan, Italy; clelia.peano@humanitasresearch.it; 7 Human Technopole, 20157 Milan, Italy

**Keywords:** *Helicobacter pylori*, CagY, B cells, T cells, cytokines, MALT, gastric lymphoma

## Abstract

Background: the neoplastic B cells of the *Helicobacter pylori*-related low-grade gastric mucosa-associated lymphoid tissue (MALT) lymphoma proliferate in response to *H. pylori*, however, the nature of the *H. pylori* antigen responsible for proliferation is still unknown. The purpose of the study was to dissect whether CagY might be the *H. pylori* antigen able to drive B cell proliferation. Methods: the B cells and the clonal progeny of T cells from the gastric mucosa of five patients with MALT lymphoma were compared with those of T cell clones obtained from five *H. pylori*–infected patients with chronic gastritis. The T cell clones were assessed for their specificity to *H. pylori* CagY, cytokine profile and helper function for B cell proliferation. Results: 22 of 158 CD4^+^ (13.9%) gastric clones from MALT lymphoma and three of 179 CD4^+^ (1.7%) clones from chronic gastritis recognized CagY. CagY predominantly drives Interferon-gamma (IFN-γ) and Interleukin-17 (IL-17) secretion by gastric CD4^+^ T cells from *H. pylori*-infected patients with low-grade gastric MALT lymphoma. All MALT lymphoma-derived clones dose dependently increased their B cell help, whereas clones from chronic gastritis lost helper activity at T-to-B-cell ratios greater than 1. Conclusion: the results obtained indicate that CagY drives both B cell proliferation and T cell activation in gastric MALT lymphomas.

## 1. Introduction

*Helicobacter pylori* is a spiral-shaped Gram-negative bacterium that chronically infects the stomach of more than 50% of the human population, and is the leading cause of gastric cancer, gastric lymphoma, gastric autoimmunity and peptic ulcer diseases [1,2,3,4,5]. A strong association between *Helicobacter pylori* infection and the development of gastric mucosa-associated lymphoid tissue (MALT) lymphoma has been demonstrated [6,7,8]. A prerequisite for lymphomagenesis is the development of secondary inflammatory MALT, which is induced by chronic *H. pylori* infection [7,8]. In the early stages, this tumor is sensitive to the withdrawal of *H. pylori*-induced T cell help, providing an explanation for both the tendency of the tumor to remain localized at the primary site and its regression after eradication of *H. pylori* with antibiotics. The tumor cells of low-grade gastric MALT lymphoma are memory B lymphocytes that still respond to differentiation signals, such as CD40 costimulation and cytokines produced by antigen-stimulated T helper (Th) cells [9,10] and their growth depends on antigen-stimulation by *H. pylori*-specific T cells [11,12]. An important unanswered question remains the chemical nature of the *H. pylori* factors responsible for the induction of gastric Th cells which can promote the proliferation of B cells. Bacterial products are known to possess immunomodulatory properties and induce B cell responses as well as different types of innate and adaptive responses [13].

Among the bacterial components, some factors associated with malignancy have been identified, although the high degree of genomic variability of *H. pylori* strains has prevented the complete identification of the factors involved. The major virulence factor of *H. pylori* is the cag pathogenicity island (cagPAI), an approximately 40 kb genetic locus, containing 31 genes [14,15] and encoding for the so-called type IV secretion system (T4SS). This forms a syringe-like structure that injects bacterial components (mainly peptidoglycan and the oncoprotein cagA) into the host target cell [16]. *H. pylori* strains harboring the cagPAI pathogenicity locus show a significantly increased ability to induce severe pathological outcomes in infected individuals, such as gastric cancer and gastric lymphoma, compared to cagPAI-negative strains [17,18,19,20]. Recently, it was reported that among *H. pylori*-infected patients, those with gastric low-grade MALT lymphoma are preferentially seropositive for *H. pylori* CagY protein [21]. CagY, a VirB10-homologous protein, also known as Cag7 or HP0527, is able to activate innate cells in a flagellin-independent manner. CagY is a TLR5 agonist and five interaction sites have been identified in the CagY repeat domains [16,22,23]. HP0527 encodes a large protein of 1927 amino acids that is expressed on the surface and has been described as one of the main components of *H. pylori* cag T4SS-associated pilum; it may act as a molecular switch that modifies the proinflammatory host responses by modulating the T4SS function and tuning CagA injection [24,25].

The aims of this study were (1) to investigate the presence of *H. pylori* CagY-specific Th cells in the context of low-grade gastric MALT lymphomas, (2) to define the cytokine patterns of these cells, and (3) to assess whether gastric CagY-specific T cells from MALT lymphomas are able to provide help for B cell proliferation.

## 2. Results

### 2.1. H. pylori CagY-Specific CD4^+^ T Cells Predominate in Gastric Low-Grade MALT Lymphoma

To characterize at the clonal level the in vivo activated T cells present in the gastric inflammatory infiltrates of *H. pylori*-infected patients, two cohorts were collected: five untreated patients with gastric low-grade MALT (MALT) and five patients with *H. pylori*-induced uncomplicated chronic gastritis (CG). All patients were infected with CagA1, VacA1 *H. pylori* type I strains and were ELISA positive for anti-CagA serum IgG antibodies. The T cells from all enrolled patients were obtained by multiple biopsies and expanded by culturing in Interleukin-2 (IL-2)-conditioned medium for 10 days. Then, T cell blasts were recovered and cloned by limiting dilution.

Comparable numbers of clones were obtained from both cohorts: a total of 158 CD4^+^ and 17 CD8^+^ clones were obtained from the gastric biopsy specimens of MALT lymphoma. 179 CD4^+^ and 22 CD8^+^ T cell clones from chronic gastritis. All clones were tested for their ability to respond to either *H. pylori* lysate or purified CagY protein. The data obtained are summarized in Table 1 and show that none of the CD8^+^ clones from MALT and CG responded to *H. pylori* CagY antigen or to *H. pylori* lysate. Analyzing the proliferative response of CD4^+^ clones to *H. pylori* lysate, 23 and 22% of the clones showed positivity for MALT and CG, respectively. A marked difference was observed when CagY was used. While 22 CD4^+^ (corresponding to 13.9%) of clones from MALT lymphoma showed antigen-induced proliferation, only three CD4^+^ clones (1.7% of the clones) from chronic gastritis were CagY-specific (Table 1).

**Figure 1:**
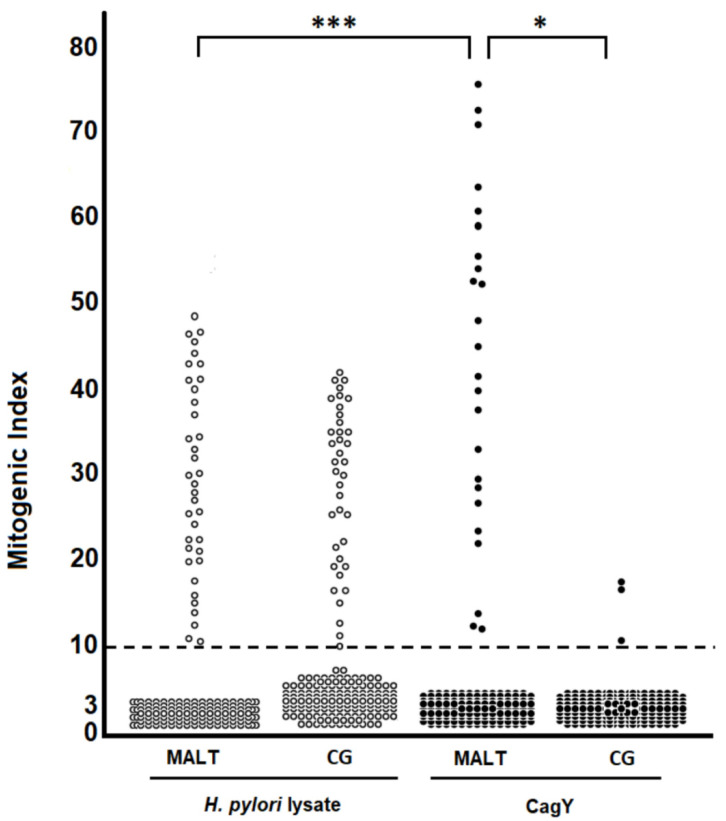
Antigen specificity of *H. pylori*-reactive T cell clones derived from the gastric mucosa of *H. pylori*-infected patients with low-grade MALT B cell lymphoma (MALT) or uncomplicated chronic gastritis (CG). In vivo activated T cells were recovered from biopsy specimens of gastric mucosa and cloned by limiting dilution. T cell blasts from each clone were seeded in triplicate cultures with irradiated autologous peripheral blood mononuclear cells in the presence of medium alone or optimal doses of *H. pylori* lysate (10 μg/mL), or CagY (1 μg/mL). After 60 h, [^3^H] thymidine uptake was measured and expressed as mitogenic index. A significant difference (*) was found between the mitogenic index of CagY-specific MALT lymphoma-derived T clones and CG-derived ones. A highly significant (***) proliferation to CagY than to H.pylori lysate was found in MALT lymphoma-derived T clones.

The percentage of MALT lymphoma-derived T clones reactive for CagY was higher than CG. A significant difference (*p* = 0.012) was also found between the mitogenic index of CagY-specific MALT lymphoma-derived T clones (mean mitogenic index 44.48 ± 18.46) and CG-derived ones (mean mitogenic index 14.63 ± 3.89). A significantly higher proliferation (*p* < 0.001) to CagY than *H. pylori* lysate was found in MALT lymphoma-derived T clones (mean CagY mitogenic index 44.48 ± 18.46; mean *H. pylori* lysate mitogenic index 29.14 ± 11.67) (Figure 1).

### 2.2. H. pylori CagY Predominantly Drives IFN-γ and IL-17 Secretion by Gastric CD4^+^ T Cells from H. pylori-Infected Patients with Gastric Low-Grade MALT Lymphoma

**Figure 2:**
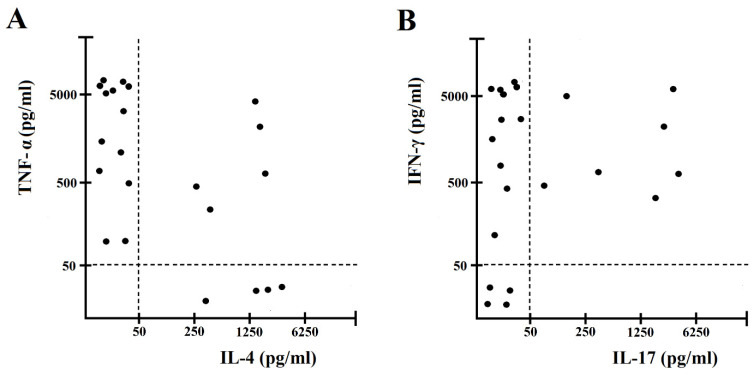
Cytokine profile of gastric mucosa CagY-specific CD4^+^ T cell clones obtained from *H. pylori*-infected patients with gastric low-grade MALT lymphoma. Th clones were tested for cytokine production (**A**,**B**). CagY-specific Th clones were stimulated with CagY and TNF-α and IL-4, IFN-γ and IL-17 production was measured in culture supernatants. In unstimulated cultures, levels of TNF-α, IL-4, IFN-γ and IL-17 were consistently < 20 pg/mL. CD4^+^ T cell clones producing IFN-γ, but not IL-17 nor IL-4, were coded as Th1. CD4^+^ T cell clones producing IL-17, but not IFN-γ nor IL-4, were coded as Th17. CD4^+^ T cell clones producing IFN-γ, and IL-17, but not IL-4, were coded as Th17/Th1. CD4^+^ T cell clones producing TNF-α and IL-4, but not IL-17, were coded as Th0.

To evaluate cytokine production by gastric-derived *H. pylori* CagY–specific Th clones, each clone was co-cultured in duplicate with autologous antigen presenting cells (APCs) and *H. pylori* CagY for 48 h. After antigen stimulation, 32% of clones from MALT lymphoma gastritis produced both Interferon-gamma (IFN-γ) and Interleukin-17 (IL-17), but not Interleukin-4 (IL-4) (Th1/Th17 profile), 27% of clones produced IFN-γ, but not IL-17, nor IL-4 (Th1 profile), 23% secreted both Tumor necrosis factor- alpha (TNF-α) and IL-4, but not IL-17 (Th0 profile), and 18% produced IL-4, but not TNF-α, nor IL-17 (Th2 profile) (Figure 2). Among the three gastric T cell clones obtained from chronic gastritis, two were Th1, and one Th1/Th17.

### 2.3. Antigen-Dependent B Cell Help by H. pylori CagY-Specific Th Clones

**Figure 3:**
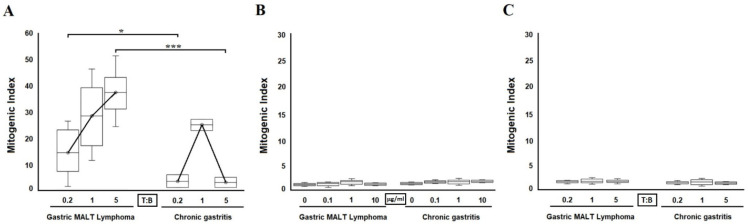
B cell proliferation to CagY. CagY-stimulated T cell clones derived from the gastric mucosa of patients with MALT lymphoma provide huge help for proliferation to autologous B cells (**A**). Irradiated T cell blasts of each CagY-reactive clone derived from patients with gastric MALT lymphoma or chronic gastritis were co-cultured for four days with peripheral blood autologous B cells (3 × 10^4^) at 0.2, 1, and 5 to 1 T-to-B-cell ratios in the presence of medium alone or CagY. Sixteen hours before harvesting, 0.5 μCi of [^3^H] thymidine was added, and its uptake was measured as mitogenic index. B cells cultured with or without CagY did not show any proliferation both in gastric low-grade MALT lymphoma and chronic gastritis patients at any CagY concentration used (0.1, 1, 10 μg/mL) (**B**). B cells cultured without CagY, with autologous irradiated gastric T cells did not show any proliferation both in gastric low-grade MALT lymphoma and in chronic gastritis patients at any T-to-B-cell ratios (0.2, 1, and 5 to 1) (**C**). * *p* < 0.05; *** *p* < 0.001.

To assess the ability of *H. pylori* CagY-specific T cell clones to provide antigen-triggered B cell help, irradiated T cell blasts of each clone were co-cultured with autologous peripheral blood B cells at a ratio of 0.2, 1, and 5 to 1. At a T-to-B cell ratio of 0.2 to 1, all CagY-specific clones from MALT lymphoma patients but none from patients with chronic gastritis, provided significant help (*p* < 0.05) to B cell proliferation under *H. pylori* CagY stimulation (mean mitogenic index, 14 and 3.5; range, 1–26 and 1–6, respectively; Figure 3A). At a T-to-B cell ratio of 1 to 1, all CagY-specific clones from both MALT lymphoma and chronic gastritis patients provided significant help for B cell proliferation under *H. pylori* CagY stimulation (mean mitogenic index, 28 and 25; range, 11–46 and 22.8–27.2, respectively; Figure 3A). Finally, at a T-to-B cell ratio of 5 to 1, all 22 Th clones from MALT lymphoma further increased B cell proliferation with a mean mitogenic index of 37 (range 24–51; Figure 3A). A significant decrease (*p* < 0.001) in B cell proliferation was observed in the presence of all three Th clones from chronic gastritis with a mean mitogenic index of 3 (range 1–5; Figure 3A). B cells alone cultured with or without CagY did not proliferate unless autologous MALT lymphoma-derived T cells were added (Figure 3B). B cells cultured with autologous MALT lymphoma-derived T cells, without CagY did not proliferate at any T-to-B cell ratio (Figure 3C).

Gastric T cell clones specific for *H. pylori* lysate but not specific for CagY (15/37 from MALT lymphoma and 36/39 from patients with chronic gastritis) showed no helper activity on B cell proliferation when co-cultured with CagY antigen and autologous B cells (mean mitogenic index was 1, range 0.9–1.3 both for MALT and for CG) (data not shown).

## 3. Discussion

*H. pylori* induces a strong inflammatory response that is directed at clearing the infection, but if not controlled, the response can be harmful to the host, and eventually lead to the development of gastric MALT lymphoma [26,27,28]. It has been shown that CagA activates the mTOR Complex 1 (mTORC1) which, in turn, promotes the expression and release of proinflammatory cytokines, chemokines, and of an antimicrobial peptide from gastric epithelial cells [29]. *H. pylori* stimulates macrophages, both in vitro and in vivo, to produce the proliferation-inducing ligand (APRIL), a crucial cytokine able to promote lymphomagenesis and B cell proliferation and abundantly expressed in gastric MALT lymphoma [30]. Lymphoma-infiltrating macrophages are a major gastric source of APRIL. By using a model of lymphomagenesis, based on the *Helicobacter* sp. infection of transgenic C57BL6 mice expressing the human form of the APRIL cytokine (Tg-hAPRIL), Blosse et al. [31] characterized the gastric mucosal inflammatory response associated with gastric MALT lymphoma and highlighted that all T cell subtypes infiltrate gastric MALT lymphoma, including regulatory T cells, both in the animal model and in human gastric MALT lymphoma patients. Regulatory T cell response might contribute to the persistence of the pathogen in the gastric mucosa by delaying the inflammatory response to allow the chronic antigen stimulation necessary for lymphoid proliferation. According to a previous report [30], authors found APRIL significantly dysregulated in human gastric MALT lymphoma and revealed that the cytokine is mainly expressed by eosinophils, suggesting the pro-tumorigenic potential of these cells. By using an antibody which recognizes the secreted and internalized form of APRIL, they also confirmed that the target cells of the cytokine are B cells [32]. Besides APRIL, also the cytokine BAFF contributes to the B cell lymphomagenesis during chronic *H. pylori* infection [31]. Chonwerawong et al. revealed that *H pylori* upregulates NLRC5 expression in the macrophages and gastric tissues of mice and humans and that this expression correlates with gastritis severity. However, by taking advantage of NLRC5-deficient macrophages, and of knockout mice with nonfunctional *Nlrc5* within the myeloid cell lineage, the authors found that NLRC5 negatively modulates the production of proinflammatory cytokines, including BAFF and protects against the formation of mucosal B cell lymphoid tissue formation in response to chronic *Helicobacter* infection in mice [33].

In vivo activated T cells in the lesional gastric mucosa of five patients with *H. pylori*–associated low-grade MALT lymphoma were expanded in our study and efficiently cloned to assess their specificity for *H. pylori* CagY protein and functional profile. This procedure has proved useful and accurate for in vitro studies of tissue-infiltrating T cells in many diseases [13,34,35]. In the progeny of gastric T cells from MALT lymphoma, but not chronic gastritis, a high proportion of T cell clones were reactive to the *H. pylori* CagY protein. Whether infection with more aggressive CagA-positive *H. pylori* strains is associated with MALT lymphoma is controversial [36,37], present data suggest that CagY is one of the immunodominant targets of gastric T cells in gastric low-grade MALT lymphoma, but not in uncomplicated chronic gastritis. On the other hand, we cannot exclude that other still undefined antigens of *H. pylori* may be involved in driving gastric T cell and B cell responses in patients with low-grade gastric MALT lymphoma.

The main limitation of this study is the small sample size enrolled, composed of five gastric MALT lymphoma patients and five chronic gastritis subjects. This is a consequence of both the fact that MALT lymphoma is not frequently found in the population and the restricted sampling caused by the difficult COVID times we are facing nowadays. This study has, however, the power to detect a relevant characterization of the cellular immune response to the *H. pylori* protein CagY given the high number of T cell clones obtained from T lymphocytes infiltrating the gastric mucosa of our tested subjects. *H. pylori* CagY-activated T cell clones from MALT lymphoma showed higher helper activity for B cell proliferation than clones generated from chronic gastritis. This supports the concept that *H. pylori*-CagY-specific T cells are responsible for the abnormal B cell growth, which probably precedes and favors the development of low-grade B cell lymphoma at gastric level in some *H. pylori*-infected patients. A possible mechanism for enhanced B cell proliferation might be the abnormal production of Th-derived cytokines active on B cell growth. MALT lymphoma-like lesions of the gastric mucosa were found after long-term *Helicobacter felis* infection in aged BALB/c mice, a strain genetically prone to high production of Th2 cytokines and B cell response [38]. Th2-skewed cytokine production in the local T cell response might account for enhanced B cell proliferation in MALT lymphoma. The majority of CagY-specific T cells in gastric MALT lymphoma produced IFN-γ and TNF-α. A significant proportion of T cells from MALT lymphoma produced IL-17 together with IFN-γ. Some CagY-specific T cells were able to produce IL-4. We can speculate that almost all CagY-specific T cells were able to produce several cytokines, such as TNF-α, and IL-4, able to promote B cell proliferation. Moreover it has been recently showed that IL-17 is also able to promote the growth of human germinal center-derived non-Hodgkin B cell lymphoma [39].

Based on the results obtained so far we can conclude that the *H. pylori* CagY protein, in patients with *H. pylori* infection and gastric low-grade MALT lymphoma, was able to promote gastric Th1 and Th17 inflammation through the production of various cytokines that can promote B cell proliferation. *H. pylori* CagY-specific Th cells derived from the gastric mucosa of *H. pylori*-infected patients with gastric low-grade MALT lymphoma were able to provide significantly higher B cell help compared to T cells obtained from patients with uncomplicated chronic gastritis.

## 4. Materials and Methods

### 4.1. Patients

Five untreated patients (three men and two women; mean age, 69 years; range, 63–75 years) with low-grade B cell lymphoma of gastric MALT (MALToma) and five patients (three men and two women; mean age, 59 years; range, 55–68 years) with uncomplicated chronic gastritis provided informed consent for this study, which was performed after approval by the local ethical committee (protocol number 14936_bio, approved on 8 October 2019). Multiple biopsy specimens were obtained from the gastric antrum of patients with chronic gastritis. In patients with low-grade MALT lymphoma, biopsy specimens were obtained from perilesional regions. Biopsy specimens were used for diagnosis (positive urease test, typing of *H. pylori* strain, and histology) and culture of tumor-infiltrating T lymphocytes. All patients with chronic gastritis or MALT lymphoma were infected with CagA1 VacA1 *H. pylori* type I strains and were positive for anti-CagA serum immunoglobulin (Ig) G antibodies, as assessed by specific ELISA (MyBioSource, San Diego, CA, USA).

### 4.2. Reagents

*Helicobacter pylori* CagY was produced as described [21]. We ruled out the presence of contaminants by a limulus test. The *H. pylori* CagY used resulted limulus test negative throughout the whole study.

### 4.3. Generation of H. pylori-Specific T Cell Clones

Biopsy specimens were cultured for 10 days in RPMI 1640 medium (Biochrom AG, Berlin, Germany) supplemented with human IL-2 (PeproTech, London, UK) (50 U/mL) to expand in vivo activated T cells [10]. Mucosal specimens were disrupted and single T-cell blasts were cloned under limiting dilution (0.3 cells/well) as reported previously [40]. Each clone was screened (in triplicate cultures for each condition) for responsiveness to *H. pylori* by measuring [^3^H] thymidine (Perkin Elmer, Waltham, MA, US) uptake after 60 h’ stimulation with medium, *H. pylori* lysate (aqueous extract of NCTC11637 strain, 10 μg/mL being optimal), recombinant CagY protein (1 μg/mL) in the presence of irradiated autologous mononuclear cells as APCs [40]. A mitogenic index greater than 10 was considered a positive result.

### 4.4. Cytokine Profile of H. pylori CagY–Specific Gastric T Cell Clones

To assess the cytokine production of CagY-specific Th clones, 10^6^ T cell blasts of each clone were co-cultured in duplicate cultures for 48 h in 1 mL of medium with 5 × 10^5^ irradiated autologous peripheral blood mononuclear cells as APCs and CagY (1 μg/mL). To induce cytokine production by gastric T cell clones, T cell blasts were stimulated for 36 h with phorbol-12-myristate 13-acetate (PMA, 10 ng/mL) (BioLegend, San Diego, CA, USA) plus anti-CD3 monoclonal antibody (200 ng/mL) (BioLegend, San Diego, CA, USA) [10]. Duplicate samples of each supernatant were assayed by ELISA for IFN-γ, IL-4, TNF-α, and IL-17 (R&D Systems, Minneapolis, MN, USA).

### 4.5. Helper Activity of T Cell Lones for B Cell Proliferation

B cells were prepared from each patient as described [13]. Briefly, PBMCs isolated by Ficoll Hypaque gradient method (Lymphoprep, Alere Technologies, Oslo, Norway) from each enrolled patient were processed to obtain a purified population of B lymphocytes by positive selection magnetic labelling with anti-CD19 microbeads (MACS, Miltenyi biotec, Bergisch Gladbach, Germany). The target population was incubated with specific microbeads and the cell suspension was loaded onto MACS columns placed in a magnetic field; B cells were then retained within the column and the other PBMCs were eluted away. Purified B lymphocytes were then harvested, washed, and used for the subsequent experiments.

The ability of gastric Th clones to induce B cell proliferation under CagY stimulation was assessed by measuring [^3^H] thymidine uptake by peripheral blood B cells (3 × 10^4^) alone or co-cultured for four days with different concentrations of irradiated (2000 rad) autologous clonal T cell blasts (0.2, 1, and 5 T-to-B cell ratio) with or without *H. pylori* CagY antigen (1 μg/mL), as described previously [10].

### 4.6. Statistical Analysis

Descriptive statistics were used for the calculation of absolute frequencies and percentages of qualitative data, as well as for mean and standard deviation of quantitative data. After evaluating the homogeneity of variance with Hartley’s test, the *t*-test (IC 95%) was performed and a *p* < 0.05 was considered statistically significant. Statistical analysis was computed using the IBM SPSS Statistics software, version 27.

## 5. Conclusions

The results obtained so far suggest that *H. pylori* CagY is an important factor involved in the genesis of Th1 and Th17 response in *H. pylori*-infected patients with gastric MALT lymphoma. We show that *H. pylori* CagY-specific Th cells derived from the gastric mucosa of *H. pylori*-infected patients with gastric low-grade MALT lymphoma can provide B cell help in a dose-dependent manner. Taken together, these results suggest that *H. pylori* CagY is an important factor in generating Th1 and Th17 responses in *H. pylori*-infected patients with gastric MALT lymphoma and that the T cell-dependent B cell proliferation induced by *H. pylori* CagY may represent an important link between bacterial infection and gastric lymphoma. CagY, Th1 and Th17 pathways might be useful for the design of novel diagnostics, novel vaccines and therapeutics for gastric MALT lymphomas related to *H. pylori* infection.

## Figures and Tables

**Table 1:** Number (%) of CagY or *H. pylori* lysate-specific T cell clones isolated from biopsy specimens of MALT lymphoma (MALT) and chronic gastritis (CG) patients.

Patient ID	CagY-Specific T CD4^+^ Clones	*H. pylori* Lysate-Specific T CD4^+^ Clones	CagY-Specific T CD8^+^ Clones	*H. pylori* Lysate-Specific T CD8^+^ Clones
MALT 1	4/30 (13.3)	5/30 (16.7)	0/3 (0)	0/3 (0)
MALT 2	5/28 (17.9)	7/28 (25)	0/4 (0)	0/4 (0)
MALT 3	5/35 (14.3)	8/35 (22.9)	0/2 (0)	0/2 (0)
MALT 4	4/22 (18.2)	7/22 (31.8)	0/4 (0)	0/4 (0)
MALT 5	4/43 (9.3)	10/43 (23.2)	0/4 (0)	0/4 (0)
Total	22/158 (13.9)	37/158 (23.4)	0/17 (0)	0/17 (0)
CG 1	1/37 (2.7)	6/37 (16.2)	0/3 (0)	0/3 (0)
CG 2	1/28 (3.6)	8/28 (28.6)	0/4 (0)	0/4 (0)
CG 3	0/35 (0)	9/35 (25.7)	0/5 (0)	0/5 (0)
CG 4	1/44 (2.3)	7/44 (15.9)	0/5 (0)	0/5 (0)
CG 5	0/35 (0)	9/35 (25.7)	0/5 (0)	0/5 (0)
Total	3/179 (1.7)	39/179 (21.8)	0/22 (0)	0/22 (0)

## Data Availability

Data supporting reported results are available from the coordinator of the study, Mario Milco D’Elios, corresponding author.
